# Parallel homodimer structures of the extracellular domains of the voltage-gated sodium channel β4 subunit explain its role in cell–cell adhesion

**DOI:** 10.1074/jbc.M117.786509

**Published:** 2017-06-27

**Authors:** Hideaki Shimizu, Asako Tosaki, Noboru Ohsawa, Yoshiko Ishizuka-Katsura, Shisako Shoji, Haruko Miyazaki, Fumitaka Oyama, Takaho Terada, Mikako Shirouzu, Shun-ichi Sekine, Nobuyuki Nukina, Shigeyuki Yokoyama

**Affiliations:** From the ‡ RIKEN Systems and Structural Biology Center, Tsurumi, Yokohama 230-0045, Japan,; the § RIKEN Center for Life Science Technologies, Tsurumi, Yokohama 230-0045, Japan,; the ¶ Laboratory for Structural Neuropathology, RIKEN Brain Science Institute, Wako, Saitama 351-0198, Japan,; the ‖ Department of Neuroscience for Neurodegenerative Disorders, Juntendo University Graduate School of Medicine, Tokyo 113-8421, Japan,; the ** Laboratory of Structural Neuropathology, Doshisha University Graduate School of Brain Science, 1–3 Tatara Miyakodani, Kyotanabe-shi, Kyoto 610-0394, Japan,; the ‡‡ Department of Chemistry and Life Science, Kogakuin University, Hachioji, Tokyo 192-0015, Japan, and; the §§ RIKEN Structural Biology Laboratory, Tsurumi, Yokohama 230-0045, Japan

**Keywords:** cell adhesion, immunoglobulin-like domain, membrane protein, sodium channel, X-ray crystallography, Navβ4, SCN4B, dimer

## Abstract

Voltage-gated sodium channels (VGSCs) are transmembrane proteins required for the generation of action potentials in excitable cells and essential for propagating electrical impulses along nerve cells. VGSCs are complexes of a pore-forming α subunit and auxiliary β subunits, designated as β1/β1B–β4 (encoded by *SCN1B–4B,* respectively), which also function in cell–cell adhesion. We previously reported the structural basis for the *trans* homophilic interaction of the β4 subunit, which contributes to its adhesive function. Here, using crystallographic and biochemical analyses, we show that the β4 extracellular domains directly interact with each other in a parallel manner that involves an intermolecular disulfide bond between the unpaired Cys residues (Cys^58^) in the loop connecting strands B and C and intermolecular hydrophobic and hydrogen-bonding interactions of the N-terminal segments (Ser^30^-Val^35^). Under reducing conditions, an N-terminally deleted β4 mutant exhibited decreased cell adhesion compared with the wild type, indicating that the β4 *cis* dimer contributes to the *trans* homophilic interaction of β4 in cell–cell adhesion. Furthermore, this mutant exhibited increased association with the α subunit, indicating that the *cis* dimerization of β4 affects α–β4 complex formation. These observations provide the structural basis for the parallel dimer formation of β4 in VGSCs and reveal its mechanism in cell–cell adhesion.

## Introduction

Voltage-gated sodium channels are transmembrane proteins responsible for the generation of action potentials in excitable cells and play important roles in the propagation of electrical impulses throughout nerves (1). They are complexes of a pore-forming α subunit and auxiliary β subunits, designated β1/β1B–β4 (encoded by *SCN1B–4B*). The β1–4 subunits, except for the secreted form of β1B, are type I, single-pass transmembrane proteins (2). The extracellular domain is structurally homologous to the V type of the Ig superfamily. The β1 and β3 subunits have four conserved Cys residues in their extracellular domains for the formation of two intramolecular disulfide bonds and are noncovalently associated with the α subunit. In contrast, β2 and β4 have three conserved Cys residues forming one intramolecular disulfide bond, and the unpaired Cys residue (Cys^55^ for β2 and Cys^58^ for β4) interacts with the α subunit via an intermolecular disulfide bond. Site-directed mutagenesis studies revealed that several negatively charged residues located in the N-terminal segments are also required for the interaction with α subunits (3–5).

In addition to the channel modulation, the β subunits function as cell adhesion molecules. The extracellular domains of the β1, β2, and β4 subunits undergo homophilic interactions in cell–cell adhesion, probably around the junctions of the nodes of Ranvier and axon initial segments with astrocytes and sheaths of Schwann (2). Furthermore, the cell adhesion activities of the β subunits are important for the regulation of neuronal migration, pathfinding, and fasciculation (6). Mutations in the extracellular domains are linked to numerous diseases, including epilepsy, sudden death syndromes, and cardiac arrhythmia, suggesting that the cell adhesion activities mediated by the extracellular domains are clinically relevant (2, 7).

**Figure 1.**
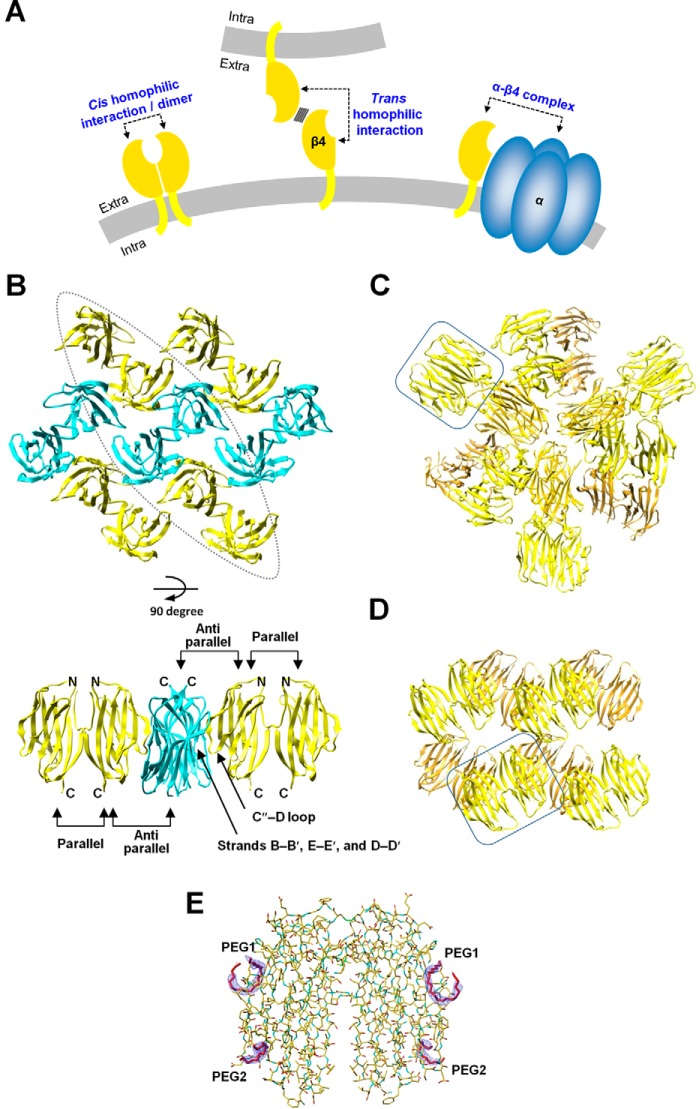
**Arrangements of the mouse/human β4 subunit extracellular domain molecules in the monoclinic, cubic, and hexagonal crystal forms.** *A*, schematics of the *cis* and *trans* homophilic interactions of β4 and the α–β4 complex. The plasma membranes are colored *gray*. The *cis* homophilic interaction of β4 and the α–β4 complex occur on the same cell. Additionally, β4 can form a *trans* homophilic interaction between two different cells. *B*, arrangement of the mouse β4 molecule in the monoclinic crystal form (PDB code 5AYQ). The β4 molecules are colored *yellow* and *cyan*. Molecules with the same color are parallel to each other, and the *yellow* molecules are oriented anti-parallel to the *cyan* ones. Shown are a top view (*top panel*) and side view of the enclosed region (*bottom panel*). *C* and *D*, arrangements of the mouse β4 molecules in the cubic crystal form (*C*) and the human β4 molecules in the hexagonal crystal form (*D*). One of the pairs of β4 molecules interacting with each other in the parallel arrangement is enclosed. *E*, electron densities of the polyethylene glycol fragments. Shown is a 2*F*_o_-*F*_c_ composite omit map (contoured at 1.5 σ) of the polyethylene glycol fragments (PEG1 and PEG2) in the hexagonal form.

We previously reported the crystal structure of the mouse β4 extracellular domain fragment (β4ex) containing all of the Cys residues (8). This structure exhibited an interface for an anti-parallel homophilic interaction in the crystal lattice. A site-directed photo-cross-linking analysis and a cell aggregation assay based on the crystal structure revealed that the interface of the anti-parallel arrangement of β4 corresponds to its *trans* homophilic interaction (Fig. 1*A*) for multimeric assembly in cell–cell adhesion. Furthermore, the *trans* interaction mode is also employed in β1-mediated cell–cell adhesion. The β1 gene mutations associated with generalized epilepsy with febrile seizures plus (GEFS+)^2^ are involved in the corresponding interface of β1 and could disrupt the *trans* homophilic interaction of β1 in cell–cell adhesion.

Our recent study revealed that the *cis* homophilic dimer of β4 (Fig. 1*A*) can be formed through disulfide bonding and is significantly abundant in unmyelinated axons of striatal projection fascicles compared with other brain regions (9). It is possible that the *cis* homophilic dimer of β4 exists and functions as a cell adhesion molecule in these neurons and that it plays a role in the fasciculation and pathfinding of striatal projection fibers. However, the structure of the *cis* homophilic dimer of the β subunit in cell–cell adhesion is unknown. In fact, the human β4ex (C58A or C131W mutants) and the human β2ex (C55A or C55A/C72A/C75A mutants) proteins exist as monomers in the crystal asymmetric units (10, 11). The crystal structure of the human β3ex revealed that the extracellular domain forms a trimer for a *cis* homophilic interaction (12), although β3 reportedly does not mediate the *trans* homophilic interaction (13).

In this study, we determined the crystal structures of the mouse and human β4ex proteins containing all three Cys residues, which revealed the formation of a dimer in a parallel arrangement, mediated by the intermolecular disulfide bond and the exchange of the N-terminal segments. A biochemical analysis demonstrated that the recombinant β4ex protein forms a dimer in solution, and a cell biological analysis revealed that the full-length β4 also forms a *cis* dimer on the cell surface of CHO cells (Fig. 1*A*). An N-terminally deleted β4 mutant exhibited decreased cell–cell adhesion under reducing conditions, indicating that the *cis* dimer formation of β4 contributes to the *trans* homophilic interaction of β4 in cell–cell adhesion (Fig. 1*A*). However, the crystal structures showed that the residues relevant to the α subunit interaction are buried within the interface in the β4 dimer, and the N-terminal-deleted β4 mutant exhibited increased association with the α subunit Nav1.5. These observations indicate that the *cis* dimerization of β4 could decrease formation of the α–β4 complex (Fig. 1*A*). This study reveals the structural basis for the parallel homophilic dimerization of β4 in cell–cell adhesion.

## Results

### Parallel arrangement of the β4 extracellular domain

In a previous study, we determined the crystal structure of the mouse β4ex protein, containing all of the Cys residues, in the monoclinic crystal form at 1.7-Å resolution (8). The crystal packing included an anti-parallel contact between the β4ex molecules that was found to correspond to the *trans* homophilic interaction in cell–cell adhesion (Fig. 1, *A* and *B*). In this study, a slightly different all-Cys construct of the mouse β4ex protein with a C-terminal tag sequence was prepared. This sample was crystallized under different conditions than those employed in the previous analysis, and the crystal structure was determined in the cubic crystal form at 3.1-Å resolution. This crystal form did not exhibit the anti-parallel arrangement but, instead, showed a parallel arrangement between the two mouse β4ex molecules, related by non-crystallographic 2-fold symmetry, in the asymmetric unit (Fig. 1*C*). The same parallel arrangement was also observed in the previous monoclinic crystal form in addition to the anti-parallel arrangement. One mouse β4ex molecule interacts with three other β4ex molecules: one in parallel and two in anti-parallel manners (Fig. 1*B*). In this study, the human β4ex containing all of the Cys residues was prepared, and its crystal structure was determined in the hexagonal form at 2.2-Å resolution. The human β4ex also assumes the same parallel arrangement as those in the previous and present mouse β4ex crystals (Fig. 1*D*). Therefore, it appears that the parallel arrangement of β4 is a conserved natural property. The anti-parallel arrangement was not observed in the cubic and hexagonal crystal forms (Fig. 1, *C* and *D*), probably because PEG fragments were bound to the *trans* homophilic interface (hexagonal form) (Fig. 1*E*) and because detergent was used for the crystallization and the C-terminal tag sequence was present (cubic form) (see “Experimental Procedures”).

**Figure 2.**
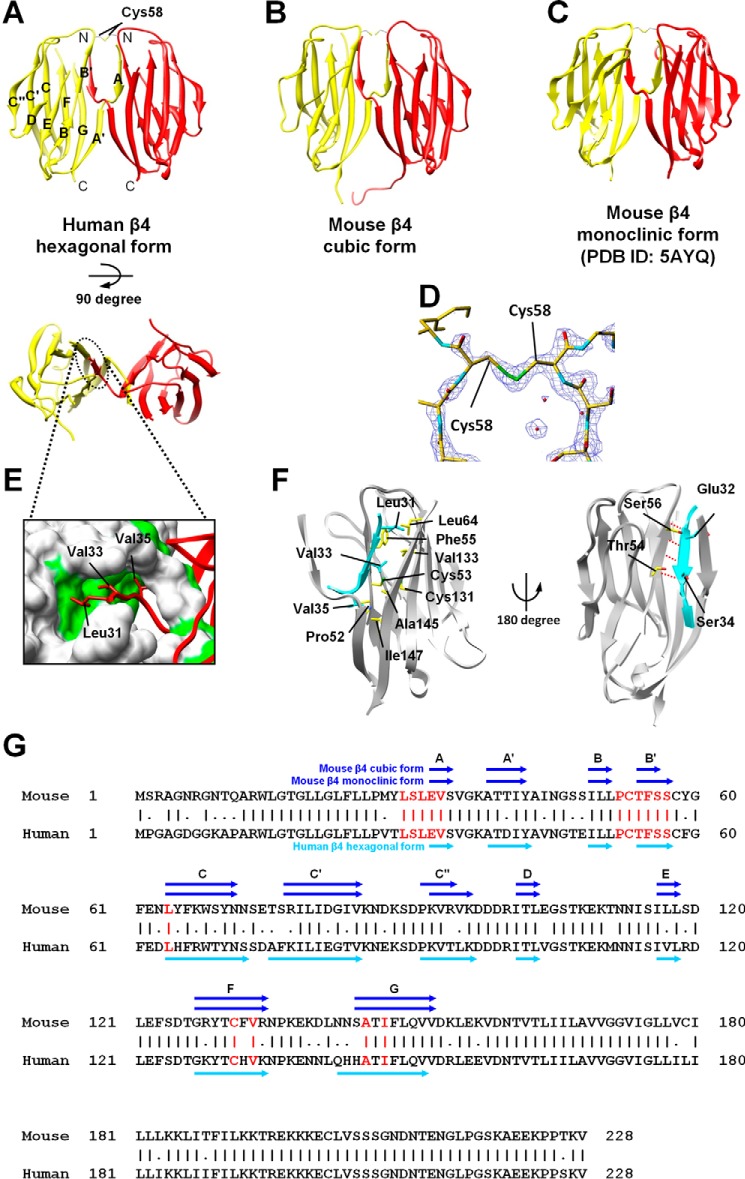
**Structures of the parallel dimer of β4.** *A–C*, ribbon representations of the β4 parallel dimer in which one molecule is colored *yellow* and the other is *red*. Shown are a side view (*top panel*) and top view (*bottom panel*). Also shown are the human β4 hexagonal form (*A*), the mouse β4 cubic form (*B*), and the mouse β4 monoclinic form (*C*). *D*, 2*F*_o_-*F*_c_ composite omit map (contoured at 1.5 σ) around the intermolecular disulfide bond between the Cys^58^ residues. *E*, the intermolecular hydrophobic interactions of the N-terminal segment (the N–N interaction). The surface-exposed hydrophobic residues are colored *green. F*, detailed views of the N–N interactions. The N-terminal segment of one molecule is colored *cyan*, and the other molecule is shown by a *gray ribbon* and a *yellow stick* model. Hydrogen bonds are shown with *dotted red lines. G*, amino acid sequences of the mouse and human β4s. The intermolecular interactions (shown in *red*) are conserved in the human and mouse β4s. The β strands are shown as *arrows*. Sequence alignment was performed using the Genetyx software (Genetyx Corp., Tokyo, Japan).

The parallel arrangement of the β4 molecules was mediated, in common, through two types of interactions (Fig. 2, *A–C*). First, the β4ex structure forms the intermolecular disulfide bond (the S–S interaction) between the “unpaired” Cys residue (Cys^58^) in the loop connecting strands B and C (Fig. 2, *A–D*). Second, the N-terminal segment Ser^30^-Val^35^, which is deleted in another structure of β4ex (10), is involved in an intermolecular interaction between the two β4ex molecules in the parallel arrangement. This type of interaction is designated hereafter as the N–N interaction. The hydrophobic side chains of Leu^31^, Val^33^, and Val^35^ of one molecule are inserted within their respective pockets formed by those of {Phe^55^, Leu^64^, and Val^133^}, {Cys^53^, Cys^131^, Val^133^, and Ala^145^}, and {Pro^52^ and Ile^147^}, respectively, in the other molecule, and *vice versa* (Fig. 2, *A–C*, *E*, and *F*, and supplemental Fig. S1). Moreover, in this N–N interaction, the N-terminal segments form hydrogen bonds between their Glu^32^ and Ser^34^ with Ser^56^ and Thr^54^, respectively, and join the β sheets of the counterparts (Fig. 2, *A–C*, *E*, and *F*). As these intermolecular interactions are sophisticated and conserved in the human and mouse β4 structures (Fig. 2*G*), we hereafter designate the two β4ex molecules in the parallel arrangement as the parallel dimer.

### Chromatographic analysis of the S–S interaction

**Figure 3.**
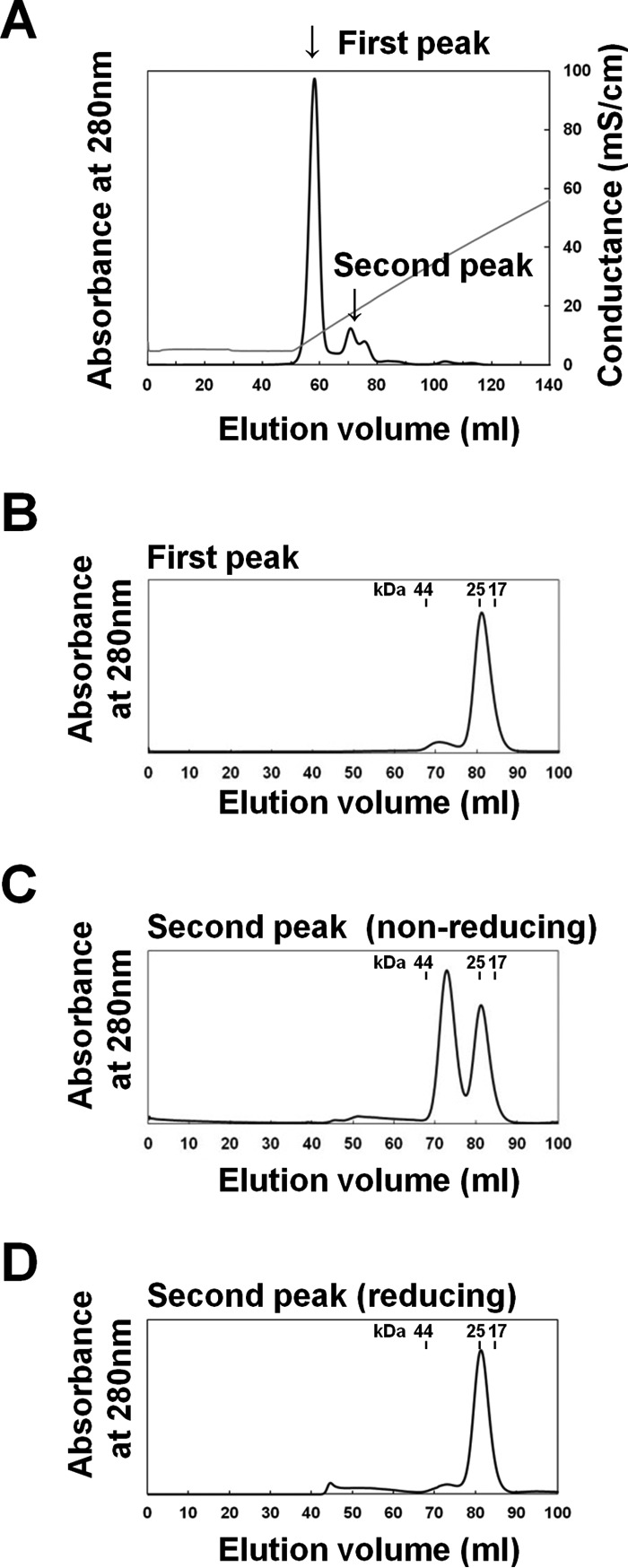
**Chromatographic analysis of the parallel dimer formation mediated by the S–S interaction.** *A–D*, elution profiles of the human β4ex protein using anion exchange chromatography under non-reducing conditions (*A*), the size exclusion chromatography (HiLoad 16/60 Superdex 75 column) for the first peak from the anion exchange chromatography under non-reducing conditions (*B*), the second peak under non-reducing conditions (*C*), and reducing conditions (*D*).

The elution profiles of the recombinant human β4ex protein from anion exchange chromatography, followed by size exclusion chromatography (HiLoad 16/60 Superdex 75 column) under non-reducing conditions, are shown in Fig. 3, *A* and *B*. The β4ex protein was eluted as two peaks (50–65 and 65–75 ml) from the anion exchange chromatography column (Fig. 3*A*). In the size exclusion chromatography, the first peak fractions at 50–65 ml were eluted at the monomer size (Fig. 3*B*), and the second peak fractions at 65–75 ml were eluted as two peaks at the monomer and dimer sizes (Fig. 3*C*). Thus, the first and second peaks from the anion exchange chromatography seem to correspond to the monomer and dimer, respectively. Moreover, the second peak fractions were eluted as a single peak at the monomer size in the presence of a reducing agent (Fig. 3*D*). Thus, the dimerization in the second fraction is mediated by the S–S interaction, and it is likely that the S–S interaction proceeds at a faster rate compared with that of the N–N interaction.

### Chromatographic analysis of the N–N interaction

**Figure 4.**
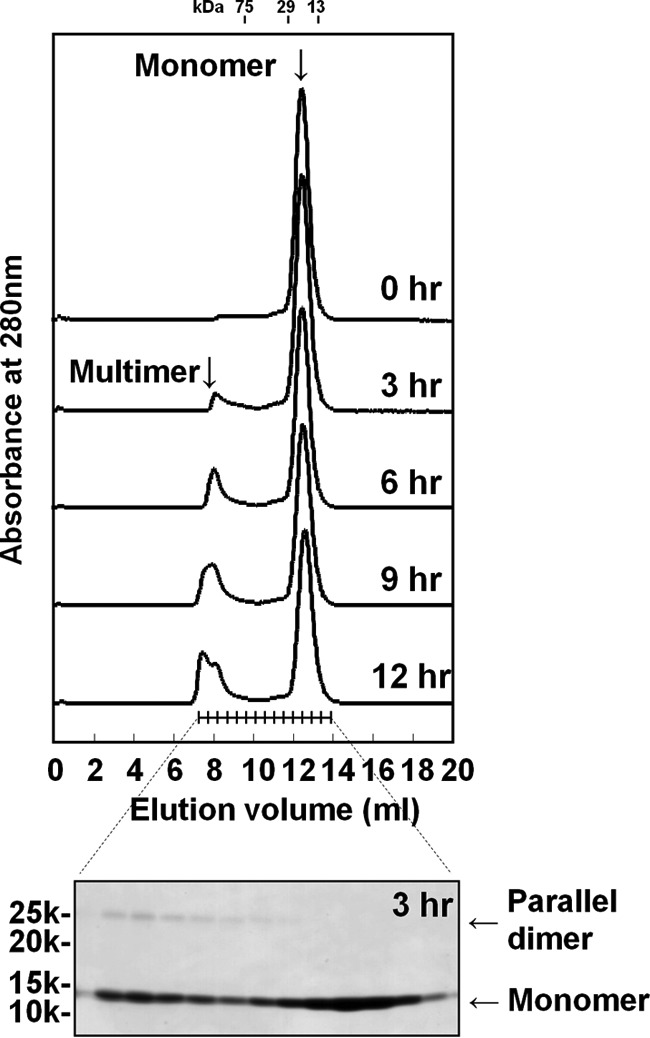
**Chromatographic analysis of the parallel dimer formation mediated by the N–N interaction.** Shown are size exclusion chromatography (Superdex 75 10/300 GL column) elution profiles of the human β4ex after 0–12 h of incubation at 20 °C (*top panel*) and SDS-PAGE analysis of the fractions eluted from size exclusion chromatography after 3 h of incubation (*bottom panel*). The incubation was performed under non-reducing conditions, and the chromatography and SDS-PAGE analysis were performed under reducing conditions.

To confirm the rate of the N-N interaction, the monomeric fraction of the human β4ex protein was incubated under non-reducing conditions at 20 °C and analyzed by size exclusion chromatography (Superdex 75 10/300 GL column) under reducing conditions. At 0 h, the β4ex protein existed predominantly as a monomer. After 3 h of incubation, a “multimer” peak appeared in addition to the monomer peak, and its intensity increased with longer incubations (Fig. 4). An SDS-PAGE analysis under reducing conditions showed that this multimer fraction actually contains the SDS-resistant dimer, which seems to be the parallel dimer with the N–N interaction (Fig. 4). It is likely that the monomeric β4ex protein converts into the parallel dimer and then further assembles into the multimer. As described above, the β4ex structure of the monoclinic crystal form exhibited the molecular layer including the anti-parallel and parallel arrangements of the β4 molecules (Fig. 1*B*) (8). The multimer formation may correspond to the combination of the anti-parallel and parallel arrangements. Notably, after 12 h of incubation, significant amounts of monomeric β4 remained, and only a small amount of β4 was dimerized. Presumably, formation of the N–N interaction continues further. In contrast, the N–N interaction was not observed when the incubation was performed at 4 °C. Therefore, the N–N interaction occurs very slowly.

### Effects of the parallel dimer formation on the trans homophilic interaction in cell–cell adhesion

**Figure 5.**
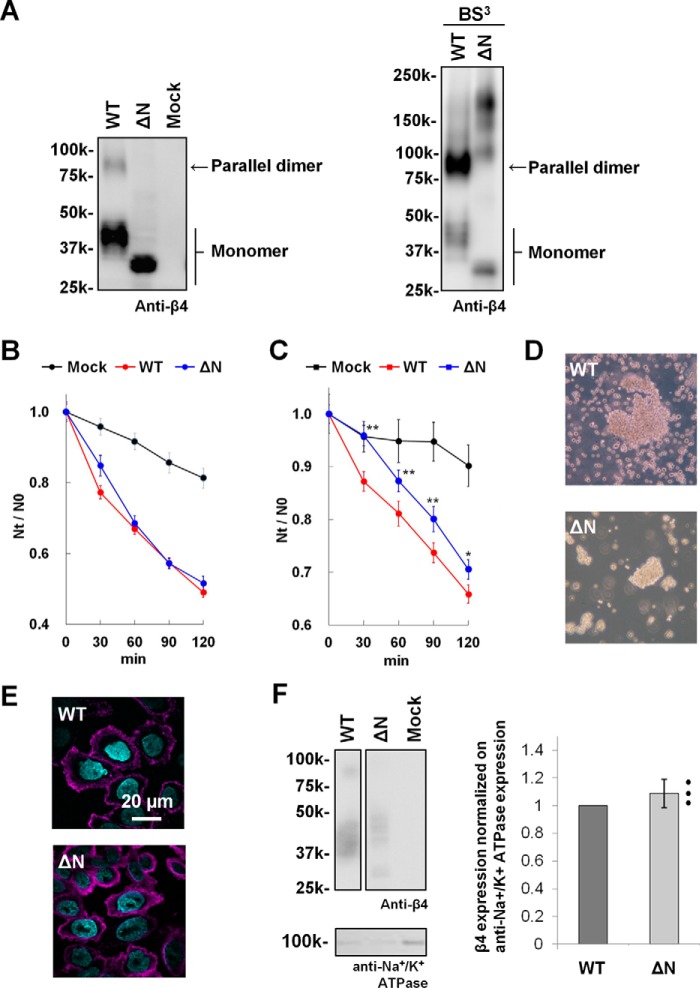
**Aggregation assay of ΔN-expressing cells.** *A*, Western blot analysis of CHO cells stably expressing the WT and ΔN mutant of mouse β4. CHO cells were treated without (*left panel*) and with BS^3^ (*right panel*). Blots were probed with anti-β4. *B*, cell aggregation kinetics of CHO cells stably expressing the WT and ΔN mutant of β4 under non-reducing conditions. Aggregation is represented by the index *N_t_*/*N*_0_, where *N_t_* and *N*_0_ are the total number of particles at incubation times *t* and 0, respectively. Data are means ± S.E., *n* = 24, from three independent experiments. Mock (*black*), WT (*red*), and ΔN (*blue*). *C*, cell aggregation kinetics under reducing conditions. Data are means ± S.E., *n* = 45, from five independent experiments. *, *p* < 0.05; **, *p* < 0.01 (two-tailed unpaired Student's *t* test) compared with the WT. *D*, cell aggregation patterns of CHO cells stably expressing the WT and mutants of β4 under reducing conditions after a 120-min incubation. Shown are the WT (*top panel*) and ΔN (*bottom panel*). *E*, immunocytochemistry of CHO cells stably expressing the WT (*top panel*) and ΔN mutant (*bottom panel*) of β4. The β4 proteins were immunostained with anti-β4 and visualized with Alexa Fluor 647 (*magenta*), and nuclei were stained with Hoechst 33342 (*cyan*). *F*, cell surface biotinylation of CHO cells stably expressing the WT and ΔN mutant of β4. Shown are representative Western blots using anti-β4 (*left panel*, *top*) and anti-Na^+^/K^+^ ATPase (*left panel*, *bottom*) and quantification of the β4 bands normalized by the corresponding anti-Na^+^/K^+^ ATPase bands (*right panel*). Data are means ± S.D., *n* = 3, and individual data are shown in scatterplots.

To explore the effects of parallel dimer formation on the *trans* homophilic interaction of β4 in cell–cell adhesion, a cell aggregation assay was performed. First, we established CHO cell lines stably expressing the WT full-length β4 (mouse) protein and its mutant with a deletion of the N-terminal segment (residues 30–37) (ΔN). A Western blot analysis confirmed that the WT-expressing cells exhibited the SDS-resistant dimer whereas the ΔN-expressing cells did not (Fig. 5*A*, *left panel*). Moreover, the dimer band was strongly detected in WT-expressing cells by treating the cells with a cell-impermeable cross-linking reagent, bis(sulfosuccinimidyl)suberate (BS^3^) (Fig. 5*A*, *right panel*). Thus, β4 appears to form a parallel dimer at the cell surface of CHO cells. After treatment with BS^3^, the ΔN mutant expressed in CHO cells exhibited several higher-molecular-mass bands at 100–200 kDa, suggesting that the ΔN mutant associates with other proteins (Fig. 5*A*, *right panel*). However, the cell aggregation assay showed that the extent of the adhesive ability of the ΔN mutant was similar to that of the WT under non-reducing conditions (Fig. 5*B*). Immunocytochemical experiments revealed that the ΔN mutant was localized at the plasma membrane of CHO cells to a similar extent as the WT (Fig. 5*E*). As these images show the total expression in permeabilized cells, to judge how much protein is truly expressed on the cell surface, we further performed a cell surface biotinylation assay. The cell surface proteins were biotinylated, and then the cell lysates were incubated with immobilized avidin to capture the biotinylated proteins, followed by a Western blot analysis. A quantification analysis of the bands showed that the ΔN mutant appears to be expressed on the cell surface of CHO cells at a similar level as the WT (Fig. 5*F*, *right panel*), although the band of the ΔN mutant was much broader than that of the WT (Fig. 5*F*, *left panel*), probably because of the altered glycosylation of the ΔN mutant. Four *N*-glycosylation sites (Asn^45^, Asn^71^, Asn^113^, and Asn^142^) were predicted in the extracellular domain of the mouse β, using the NetNGlyc and NetOGlyc servers (http://www.cbs.dtu.dk/services/).^3^ Moreover, we reported previously that the β4 protein expressed in CHO cells is *N*-glycosylated (8). Thus, it is presumable that the altered glycosylation of the ΔN mutant concerns the *N*-glycosylation at these residues.

Next we performed cell aggregation assays with these cell lines. The ΔN-expressing cells showed a similar degree of cell aggregation as the WT-expressing cells under non-reducing conditions (Fig. 5*B*). However, the relative degree of cell aggregation by the ΔN-expressing cells was smaller than that of the WT-expressing cells under reducing conditions (Fig. 5, *C* and *D*). On the basis of these observations, we suggest that the ΔN mutant on the cell surface could form a parallel dimer by S–S interaction under non-reducing conditions and then become a monomeric form by the treatment with reducing agents, thereby decreasing the ability to mediate cell–cell adhesion.

### Effect of parallel dimer formation on the association of β4 with the α subunit

**Figure 6.**
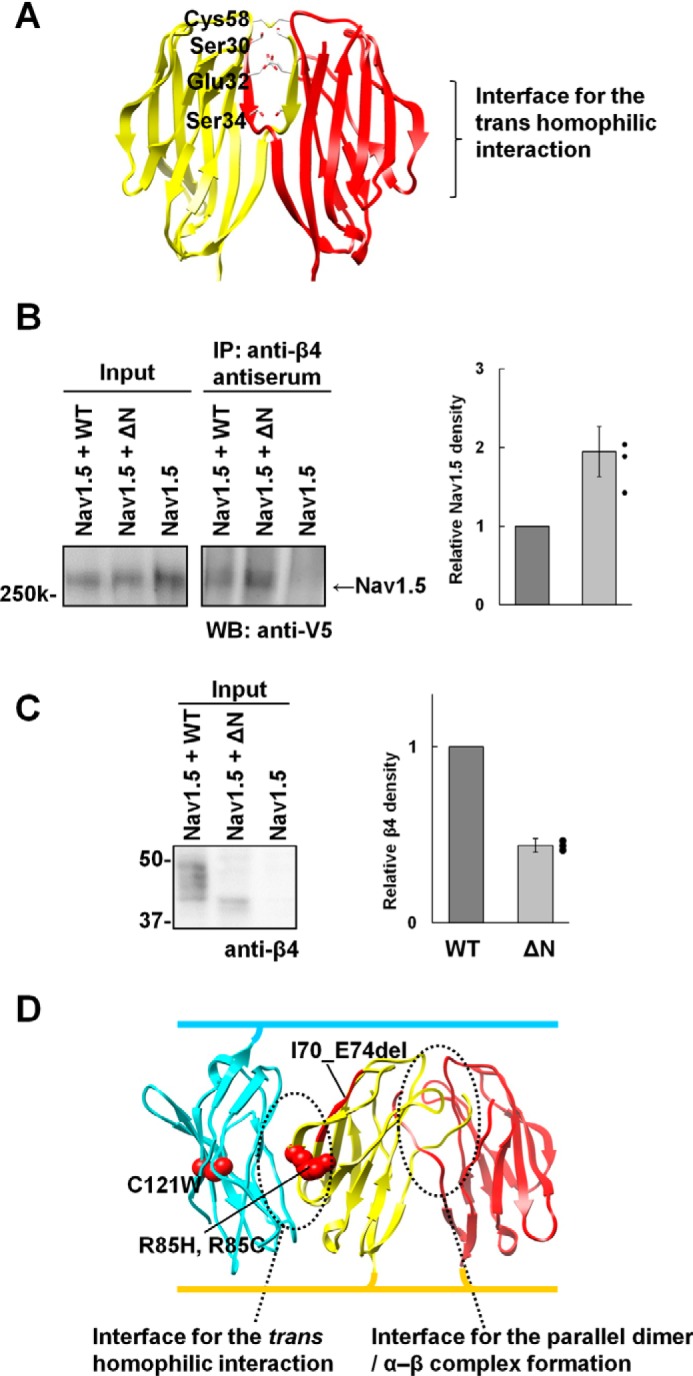
**Interface with the α subunit.** *A*, the N-terminal residues required for modulation of the α subunits are mapped onto the β4 crystal structure. *B*, immunoprecipitation of HEK293 cells transiently transfected with Nav1.5-V5-His + WT (mouse), Nav1.5-V5-His + ΔN (mouse), and Nav1.5-V5-His alone using anti-β4 antiserum. Blots were probed with anti-V5. The graph shows the Nav1.5 intensity from Nav1.5 + ΔN–transfected cells relative to that from Nav1.5 + WT–transfected cells. Data are means ± S.D. from three independent experiments, and individual data are shown in scatterplots. *C*, Western blot analysis of the input samples for the co-immunoprecipitation study, probed with anti-β4. The graph shows the β4 intensity from Nav1.5 + ΔN–transfected cells relative to that from Nav1.5 + WT–transfected cells. Data are means ± S.D. from three independent experiments, and individual data are shown in scatterplots. *D*, mapping of the GEFS+ mutations on the β1 model structure. The model was generated by the superimposition of the monomer β1 model (built by the program MODELLER (34)) onto the mouse monoclinic β4 structure.

In the case of β1, the unpaired Cys residue and the N-terminal segment interact with the α subunit (3–5). Interestingly, the crystal structures showed that the side chains of the N-terminal segment residues are located within the interface between the N–N interaction molecules and thereby unexposed on the parallel dimer surface (Fig. 6*A*). To examine whether parallel dimer formation of β4 affects α–β4 complex formation, we performed a co-immunoprecipitation study. β4 functionally binds to the cardiac α subunit isoform Nav1.5 (14, 15), and *SCN4B* mutations are associated with cardiac arrhythmias (16, 17). In addition, the association of β4 with Nav1.5 was detected in a co-immunoprecipitation study using HEK293 cells (16). Therefore, Nav1.5 was used in this study. HEK293 cells were transiently co-transfected with expression vectors encoding Nav1.5-V5-His and the WT or ΔN of mouse β4. The cell lysates were immunoprecipitated with anti-β4 antiserum and Western-blotted with an anti-V5 antibody. The Nav1.5 band was present in the immunoprecipitates from HEK293 cells co-transfected with Nav1.5-V5-His and the WT or ΔN of β4 but was not detected in HEK293 cells transfected with only the Nav1.5-V5-His (Fig. 6*B*, *left panel*). The ΔN mutant lacks the residues in the N-terminal segment required for modulation of the α subunits (3–5). Thus, the association of the ΔN mutant with Nav1.5 is presumably due to disulfide bonding between the α and β4 subunits, as reported previously (1, 15, 18). Furthermore, the ΔN mutant was found to exhibit a 2-fold association with Nav1.5 compared with the WT (Fig. 6*B*, *right panel*), although the expression level of the ΔN mutant was somewhat lower than that of the WT (Fig. 6*C*). These observations indicated that parallel dimer formation could decrease the association of β4 with Nav1.5.

## Discussion

So far, the molecular functions of β4 have been understood mainly through assumptions that β4 is present in the monomeric state. Our structural analysis provided a detailed view of the parallel dimer of β4, involving the N–N interaction between the N-terminal segments of the two β4ex molecules and the S–S interaction formed by the intermolecular disulfide bond between the unpaired Cys residues in the parallel arrangement. We also revealed that the parallel dimer formation of β4 seems to increase the *trans* homophilic interaction and decrease the α subunit association of β4. These findings suggest that the parallel dimer formation is physiologically relevant and provide new insights into the functional architecture of β4.

As shown in Figs. 3 and 4, the S–S interaction forms rapidly compared with the N–N interaction. Moreover, we observed that the monomeric β4ex protein could not readily convert into the parallel dimer under reducing conditions.^4^ These observations indicate that the S–S interaction facilitates formation of the N–N interaction. This may be consistent with the findings that the parallel dimer was not observed in the previously reported structures of the β4ex C58A mutant (10) and the β2ex C55A and C55A/C72A/C75A mutants (11).

**Figure 7.**
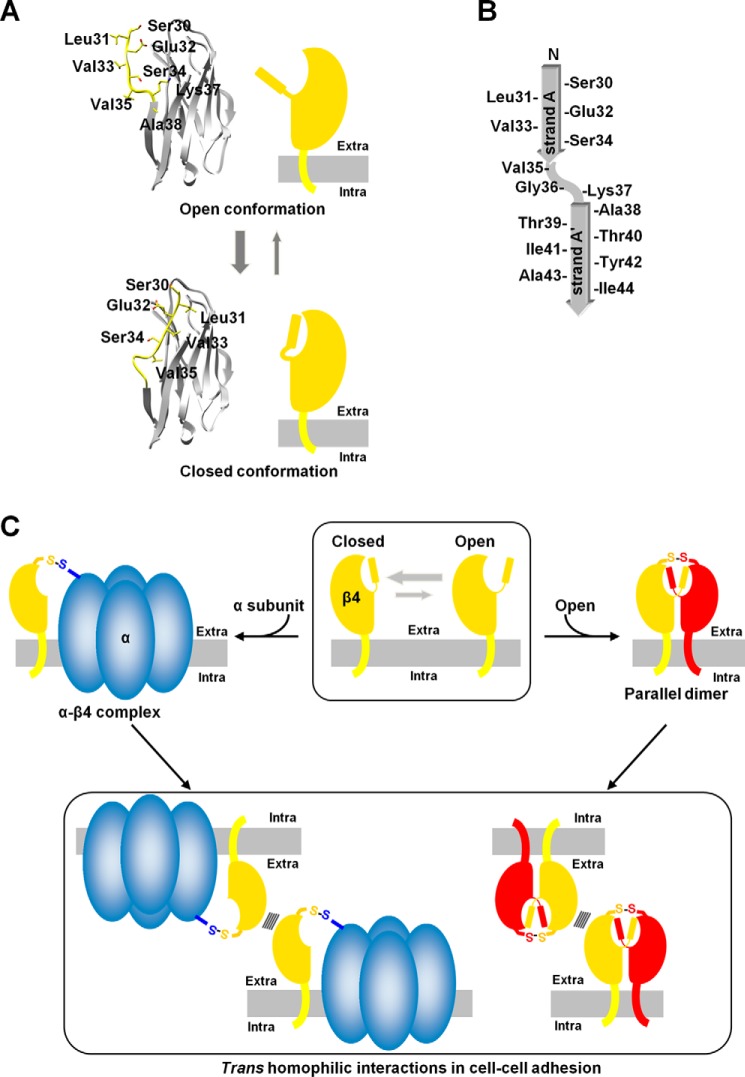
**Schematics of β4.** *A*, Open (*top panel*) and closed (*bottom panel*) conformations. The plasma membranes are colored *gray*. The β4 protein may accommodate the hydrophobic residues in its own hydrophobic pocket to form a monomer in a closed conformation. *B*, topology diagram of strand A/A′. *C*, the *trans* homophilic interactions of the parallel dimer and the monomer of β4 in cell–cell adhesion. The closed and open conformations of β4 are likely to exist in equilibrium, and the parallel dimer could be readily formed when two monomers in the open conformation meet each other. The monomeric β4 can associate with the α subunit. Both the α-β4 complex and parallel dimer could exhibit cell–cell adhesion.

In the N–N interaction, the three hydrophobic residues Leu^31^, Val^33^, and Val^35^ participate in hydrophobic interactions with the other molecule in the parallel dimer (Figs. 2, *E* and *F*). However, it is possible that the β4 protein may accommodate these residues in its own hydrophobic pocket to form a monomer in a closed conformation (Fig. 7*A*). In this conformation, the hinge region between strands A and A′ may form a small bulge-like conformation by stacking with the anchoring Val^35^ (Fig. 7, *A* and *B*). Thus, the closed and open conformations are likely to exist in equilibrium, and the parallel dimer could be readily formed when two monomers in the open conformation meet each other (Fig. 7*C*). The size exclusion chromatography analysis showed the slow process of the parallel dimer formation (Fig. 4). The relative abundance of the monomer in the open conformation is presumably smaller than that in the closed conformation.

Based on our findings, we propose a scheme involving the *trans* and *cis* homophilic interactions of β4 (Fig. 7*C*), which may apply, completely or partly, to β1 and β2 as well. The monomeric β4 undergoes the open–closed conformational equilibrium, and the open conformation is readily available for parallel dimer formation, whereas the closed conformation prevents formation of the parallel dimer with the N–N interaction. In the absence of the α subunit, β4 may predominantly form the parallel dimer and participate in cell–cell adhesion. As shown in Fig. 2*A*, β4ex exhibits a highly ordered conformation in the crystal lattice, in which two parallel dimers interact with each other in the anti-parallel arrangement as the *trans-*homophilic interaction, resulting in formation of the molecular layer. In this context, it can connect one parallel dimer with four other parallel dimers. Such multimeric interactions are likely to reinforce *trans* homophilic cell–cell adhesion, even when the individual *trans* interaction between two parallel dimers is not very strong. It is likely that this arrangement exists between adhered cells and promotes the clustering of β4 at cell–cell contacts.

The crystal structure of β4 indicated that the residues relevant to the α subunit interaction are buried at the interface of the parallel dimer (Fig. 6*A*). In addition, the monomeric ΔN mutant exhibited increased association with Nav1.5 (Fig. 6*B*). In the presence of the α subunit, it appears that the monomeric β4 can associate with the α subunit whereas the β4 parallel dimer cannot. The cell aggregation assay showed that the monomeric ΔN mutant also retained cell–cell adhesion ability (Fig. 5, *C* and *D*). As shown in Fig. 6*A*, the interface for cell–cell adhesion is located opposite to the relevant residues for the α subunit interaction. Thus, it seems likely that formation of the α–β4 complex, in which the monomeric β4 is present, could exhibit cell–cell adhesion (Fig. 7*C*), and this is in good agreement with previous reports that the α–β1 complex participates in cell–cell adhesion (19, 20).

As described above, the N-terminal region is likely to exhibit conformational flexibility in the open conformation of the monomeric state. Moreover, this region reportedly contains the residues required for modulation of the α subunits (3–5). This region may assume multiple conformations that are used differentially in interactions with the α subunit isoforms, which may lead to the α subunit isoform-specific effects of the β subunits on channel modulation. Further analyses, especially an electrophysiological analysis, will clarify the effects of the β4 monomer *versus* the parallel dimer on the channel function of the α subunit.

Our previous study suggested that disruption of the *trans* homophilic interaction was implicated in the GEFS+ mutations of *SCN1b* (8). All of the GEFS+ mutations are located in the vicinity of the interface for the *trans* homophilic interaction and are not directly involved in the interface for parallel dimer/α–β complex formation (Fig. 6*D*). It is therefore probable that the GEFS+ mutations could affect not only the α subunit association but also the parallel dimer formation if β1 could form the parallel dimer. Consequently, the GEFS+ phenotype is due to the disruption of the *trans* interactions of β1 for cell adhesion mediated by the α–β1 complex (8, 21) and the parallel dimer.

The crystal structure of the β3 extracellular domain showed the trimer conformation for the *cis* homophilic interaction (12), and β3 reportedly lacks the *trans* homophilic interaction (13). Our previous structural study suggested the involvement of the C″–D loop in the *trans* homophilic interaction for the cell–cell adhesion of β4 (8). Apparently, both the monomeric and dimeric forms of β4 enable the extracellular domains to maintain the suitable spatial arrangements of their C″–D loops for the *trans* homophilic interaction. In the trimeric form of β3, however, the C″–D loop is located within the trimeric structure and cannot interact with another β3 molecule; therefore, this form lacks the *trans* homophilic interaction.

This study provides the structural basis for the parallel dimer of β4. In our proposed scheme (Fig. 7*C*), the formations of the parallel dimer and the α–β4 complex could competitively inhibit each other's binding. In the absence of the α subunit or in the presence of abundant β4, the parallel dimer could become the dominant species. This could enhance *trans* homophilic adhesion and subsequently promote β4 clustering at cell–cell contacts. Recently, we reported that β4 exhibited a diffused distribution along the unmyelinated axons in striatal projection fibers and that the dimeric β4 proteins were significantly increased in these neurons (9). The parallel dimers of β4 may be present in these neurons and adhere to each other, but further studies are needed to verify the physiological significance of the β4 parallel dimer.

## Experimental procedures

### Recombinant protein expression by Escherichia coli and crystallization

The DNA fragment encoding residues 30–160 of the mouse β4 subunit (GenBank accession no. BK001031.1), containing a C-terminal His_6_ tag, was cloned into the pGEX6P2 expression vector (GE Healthcare) and expressed in *E. coli* strain BL21DE3 cultured in terrific broth medium (amino acid numbering starts from the initiator methionine residue). When the culture reached an *A*_600_ of 0.4–0.6, protein expression was induced by addition of 1 mm isopropyl 1-thio-β-d-galactopyranoside for 2–3 days at 17–23 °C. The harvested cells were lysed with lysozyme and DNase in 100 mm Tris-HCl buffer (pH 8.0) containing 400 mm NaCl, 20% (v/v) glycerol, 0.1% (v/v) Tween 20, and 1 mm PMSF. The cell homogenates were centrifuged at 40,000 × *g* for 1 h, and the supernatants were loaded onto a nickel-Sepharose column (all chromatography materials were purchased from GE Healthcare) and eluted with 50 mm Tris-HCl buffer (pH 7.5) containing 400 mm NaCl, 20% (v/v) glycerol, and 200 mm imidazole. The protein fractions were loaded onto a glutathione-Sepharose column and eluted with 50 mm Tris-HCl buffer (pH 7.5) containing 400 mm NaCl, 20% (v/v) glycerol, and 10 mm glutathione. The GST tag was cleaved with PreScission protease (GE Healthcare) at 4 °C for 2–3 days. Finally, the proteins were dialyzed against 50 mm Tris-HCl buffer (pH 8.0) containing 20% (v/v) glycerol, loaded onto a Mono Q column, and eluted with a linear gradient from 0–400 mm NaCl. The purified proteins were concentrated to 5–10 mg/ml.

The crystals of the mouse β4 protein (cubic form) were grown by sitting drop vapor diffusion method at 20 °C. The drops consisted of 0.5 μl of protein solution and 0.5 μl of reservoir solution containing 2.7 m sodium malonate, 0.1 m Tris-HCl (pH 7.0), and 0.5% (v/v) Tween 20.

### Protein preparation by cell-free protein synthesis method and crystallization

Protein preparation was performed as described previously (8). The DNA fragment encoding residues 30–152 of the human β4 subunit (GenBank accession no. NP_777594) was cloned into the expression vector pCR2.1 TOPO (Life Technologies) as fusions with an N-terminal His tag and a tobacco etch virus protease cleavage site. The proteins were produced by the *E. coli* cell-free method (22) and purified as described previously (8). The reaction solutions were centrifuged at 16,000 × *g* and 4 °C for 20 min, and the supernatants were loaded onto a HisTrap column equilibrated with 20 mm Tris-HCl buffer (pH 8.0) containing 1.0 m NaCl, 10% (v/v) glycerol, and 20 mm imidazole. After washing the column with the buffer, the His-tagged proteins were eluted with 20 mm Tris-HCl buffer (pH 8.0) containing 500 mm NaCl, 10% (v/v) glycerol, and 500 mm imidazole. The sample buffer was exchanged to 20 mm Tris-HCl buffer (pH 8.0) containing 1.0 m NaCl, 10% (v/v) glycerol, and 20 mm imidazole with a HiPrep 26/10 desalting column, and the His tag was cleaved by tobacco etch virus protease at 4 °C overnight. To remove the uncleaved protein, the reaction solutions were loaded onto the HisTrap column as described above. The flow-through fractions were collected and desalted on a HiPrep 26/10 desalting column equilibrated with 20 mm Tris-HCl buffer (pH 8.5) containing 20 mm NaCl and 10% (v/v) glycerol. The pooled fractions were loaded onto a HiTrap Q column equilibrated with 20 mm Tris-HCl buffer (pH 8.0) containing 10 mm NaCl and 10% (v/v) glycerol. The proteins were eluted with a linear gradient from 10 mm to 1.0 m NaCl. Finally, the purified protein fractions were loaded on a HiLoad 16/60 Superdex 75 column equilibrated with 20 mm Tris-HCl buffer (pH 8.0) containing 150 mm NaCl and 10% (v/v) glycerol. The purified proteins were concentrated to 8–11 mg/ml.

The crystals of the human β4 protein (hexagonal form) were grown by using a TERA automatic crystallization system at 20 °C (23). The crystallization drop was prepared by mixing 0.5 μl of protein solution and 0.5 μl of precipitant solution containing 10% (w/v) PEG1000 and 10% (w/v) PEG8000. The drop was overlaid with a 1:1 mixture of silicone and paraffin oils.

### Determination of the structures of β4

The cubic crystal was directly flash-cooled in the nitrogen gas stream. The hexagonal crystal was transferred to mother liquor containing 15% (v/v) glycerol as a cryoprotectant and flash-cooled in a nitrogen gas stream at 100 K. Diffraction data were collected at 100 K on BL44B2 (24) and BL26B2 via the mail-in data collection system (25) at SPring-8 Center using X-rays with wavelengths of 0.8 and 1.0 Å, respectively. All data sets were integrated and scaled using the HKL2000 and SCALEPACK software packages (26). The structures were solved by molecular replacement using the program MrBUMP in the CCP4 package (27) by employing the β4 structure determined previously (PDB code 5AYQ) as the search model. The structures were built with the program *ARP*/*wARP* (28) and refined by rigid body fitting followed by the simulated-annealing protocol implemented in the program CNS (29). Model building and further refinement were performed with the programs COOT (30) and PHENIX (31). Data collection and refinement statistics are shown in supplemental Table S1. The refined coordinates have been deposited in the PDB under accession numbers 5XAX (mouse β4 cubic form) and 5XAW (human β4 hexagonal form).

### Analytical size exclusion chromatography

The monomer fraction of the human β4ex protein, prepared as described above, was separated by chromatography on a HiLoad 16/60 Superdex 75 column and then concentrated to 9 mg/ml. After 0–12 h of incubation at 20 °C, 35 μl of the β4ex protein solution was loaded on a Superdex 75 10/300 GL column equilibrated with 20 mm Tris-HCl buffer (pH 8.0) containing 150 mm NaCl, 10% (v/v) glycerol, and 5 mm β-mercaptoethanol at 4 °C.

### Antibodies

Primary antibodies were as follows: rabbit polyclonal anti-β4 (32), mouse monoclonal anti-V5 (Life Technologies, R960-25), and mouse monoclonal anti-Na^+^/K^+^ ATPase (Abcam, ab7671). Secondary antibodies were as follows: chicken anti-rabbit IgG (H+L) Alexa Fluor 647 (Life Technologies, A21443), which was used for immunofluorescence, and HRP-conjugated anti-mouse and anti-rabbit IgG (GE Healthcare, NA931 and NA934, respectively), which were used for Western blotting.

### Cell culture and transfection

For stable expression, the Flp-In CHO cell lines expressing mouse β4 were generated as described previously (8). The DNAs were cloned into the expression vector pcDNA5/FRT and transfected into cells using Lipofectamine 2000 (Life Technologies). The transfected cells were selected with 400–500 μg/ml hygromycin. Protein expression was induced by adding 1.0 μg/ml doxycycline to the medium for 16 h, and clones expressing similar levels of β subunits were selected by limited dilution, Western blotting, and immunocytochemical analyses. For transient expression, HEK293T cells were cultured in a 1:1 mixture of DMEM and Ham's F12. The DNAs encoding mouse Nav1.5 (GenBank accession no. AK147517.1, obtained from the RIKEN mouse FANTOM cDNA library) and β4 were cloned into the expression vector pcDNA3.1/V5-His-TOPO with (Nav1.5) or without (β4) the V5-His tag, as described previously (32, 33), and transfected into cells using Lipofectamine 2000.

### Western blotting

Western blotting was performed as described previously (32, 33). The cells were washed twice with PBS and lysed in 25 mm Tris-HCl buffer (pH 8.0) containing 150 mm NaCl, 0.1% (w/v) SDS, 1.0% (v/v) Triton X-100, 0.5% (w/v) deoxycholic acid, and protease inhibitors. The supernatants, collected after centrifugation at 500 × *g* for 10 min at 4 °C, were heated at 70 °C for 3 min in lithium dodecyl sulfate sample buffer (Life Technologies) containing 5 mm DTT, fractionated on 5–20% gradient polyacrylamide gels, and probed with the indicated antibodies. Full-length blots are presented in supplemental Fig. S2.

### BS^3^ cross-linking

The cells were incubated with 1 mm BS^3^ in PBS for 30 min at room temperature. The cells were washed twice with PBS and analyzed by Western blotting.

### Cell aggregation assay

The cell aggregation assay was performed as described previously (8). The cells were grown to 70–80% confluency and induced by adding 1.0 μg/ml doxycycline to the medium for 16 h. After washing twice with PBS, the cells were incubated with PBS containing 2 mm EDTA (pH 7.5) for 15 min at 37 °C and then dispersed by gentle pipetting. The cells were suspended in DMEM at 1.0 × 10^6^ cells/ml and then transferred to polystyrene tubes. The cell suspensions were incubated at 37 °C, and aliquots were counted at 30-min intervals with a hemocytometer after mixing by several gentle inversions. For reducing conditions, the cell suspensions were treated with DMEM containing 0.5 mm DTT for 10 min at room temperature, and then the medium was replaced with DMEM containing 0.1 mm β-mercaptoethanol. The cell aggregation assay of the CHO cells co-expressing Nav1.5 and β4 was not performed, as no association of β4 with Nav1.5 was observed in the stable β4-expressing CHO cell lines transiently transfected with Nav1.5.

### Immunofluorescence microscopy

Immunofluorescence microscopy was performed as described previously (8). The cells were grown on coverslips coated with collagen and induced with doxycycline as described above. The cells were then fixed with PBS containing 4% (w/v) paraformaldehyde for 15–20 min and permeabilized with PBS containing 50 μg/ml digitonin for 2 min at room temperature. The cells were incubated with PBS containing 50 mm ammonium chloride for 5 min, blocked with PBS containing 0.1% (w/v) gelatin for 5 min, and then incubated with anti-β4 antibody for 1 h at room temperature. After the primary antibody incubation, the cells were subsequently incubated with Alexa Fluor 647-conjugated secondary antibody for 30 min at room temperature. Cell nuclei were labeled with Hoechst 33342. The cells were mounted with a SlowFade antifade kit (Molecular Probes) and viewed using a Zeiss LSM 510 confocal microscope.

### Cell surface biotinylation

The cells were biotinylated and isolated with a cell surface protein isolation kit (Pierce 89881) according to the protocol of the manufacturer. Samples were analyzed by Western blotting.

### Co-immunoprecipitation

The protein A or protein G magnetic beads (Dynal ASA and GE Healthcare, respectively) were incubated with polyclonal rabbit antiserum (9), generated against the recombinant mouse β4 protein synthesized by the cell-free protein synthesis method for crystallization, and washed extensively. HEK293 cells plated in 100-mm cell culture dishes were transfected with 12 μg of the mouse Nav1.5-pcDNA3.1/V5-His-TOPO vector (with the V5-His tag) plus 4 μg of the mouse WT- or ΔN-pcDNA3.1/V5-His-TOPO vector (without the V5-His tag). 44 h after transfection, the cells were detached from the culture dishes with 50 mm Tris-HCl buffer (pH 8.0) containing 10 mm EDTA (pH 7.5) (TE buffer) and centrifuged at 200 × *g* for 10 min at 4 °C. The cell pellets were washed with TE buffer and lysed in an appropriate volume of 60 mm Tris-HCl buffer (pH 7.5) containing 180 mm NaCl, 1.25% (v/v) Triton X-100, 6 mm EDTA (pH 7.5), and protease inhibitors. After incubation for 30 min at 4 °C, the supernatants were collected after centrifugation at 14,000 × *g* for 30 min at 4 °C and incubated with the beads by rotating end over end for 3 h at 4 °C. The beads were then washed extensively with 50 mm Tris-HCl buffer (pH 7.5) containing 150 mm NaCl, 0.1% (v/v) Triton X-100, 5 mm EDTA (pH 7.5), and protease inhibitors. The immunoprecipitated proteins were eluted with LDS sample buffer containing 5 mm DTT and analyzed by Western blotting using the indicated antibodies. Unfortunately, the Nav1.5-β4 complex was not detected under the non-reducing conditions, as it gave a poor signal and a high background.

### Statistical analysis

Two-tailed unpaired Student's *t* test was performed for comparisons between two groups of samples, as described in the figure legends. All data had normal distributions (tested by Shapiro-Wilk test and D'Agostino-Pearson omnibus test) and equal variances (tested by *F* test and Bartlett test).

## Author contributions

H. S., H. M., F. O., N. N., and S. Y. designed the research. H. S., Y. I. K., N. O., S. Shoji, and A. T. performed the experiments. T. T. and M. S. helped with the protein preparations. H. S., S. Sekine, N. N., and S. Y. analyzed the data. H. S. and S. Y. wrote the paper.

## Supplementary Material

Supplemental Data

## Footnotes

3 Please note that the JBC is not responsible for the long-term archiving and maintenance of this site or any other third party–hosted site.

4 H. Shimizu, unpublished data.

## Footnotes

2 The abbreviations used are:

GEFS+: generalized epilepsy with febrile seizures plus
BS^3^: bis(sulfosuccinimidyl)suberate.
