# Supplementary material for: Parallel homodimer structures of the extracellular domains of the voltage-gated sodium channel β4 subunit explain its role in cell–cell adhesion
Source: J Biol Chem. 2017 Jun 27;292(32):13428–40. doi: 10.1074/jbc.M117.786509 (PMC5555201; doi:10.1074/jbc.M117.786509)
Supplement: Supplemental Data [file supp_292_32_13428__index.html]

Parallel homodimer structures of the extracellular domains of the voltage-gated sodium channel β4 subunit explain its role in cell-cell adhesion — Parallel homodimer structures of the extracellular domains of the voltage-gated sodium channel β4 subunit explain its role in cell–cell adhesion — Parallel homodimer structures of Navβ4 — Supplemental Data 

# Parallel homodimer structures of the extracellular domains of the voltage-gated sodium channel β4 subunit explain its role in cell–cell adhesion

## Supplemental Data

- Supplemental data (.pdf, 3.5 MB) - Supplemental Figure S1, S2 and Supplemental Table S1
