# Supplementary material for: Parallel homodimer structures of the extracellular domains of the voltage-gated sodium channel β4 subunit explain its role in cell–cell adhesion
Source: J Biol Chem. 2017 Jun 27;292(32):13428–40. doi: 10.1074/jbc.M117.786509 (PMC5555201; doi:10.1074/jbc.M117.786509)
Supplement: Supplemental Data [file 10.1074_M117.786509_jbc.M117.786509-1.pdf]

SUPPLEMENTAL DATA

Parallel homodimer structures of the extracellular domains of the voltage-gated sodium channel β4 subunit explain its role in cell-cell adhesion

Hideaki Shimizu[1,2,3], Asako Tosaki[3], Noboru Ohsawa[1,2], Yoshiko Ishizuka-Katsura[1,2], Shisako Shoji[1,2], Haruko Miyazaki[3,4,5], Fumitaka Oyama[3,6], Takaho Terada[1,7], Mikako Shirouzu[1,2], Shun-ichi Sekine[1,2], Nobuyuki Nukina[3,4,5] and Shigeyuki Yokoyama[1,7]

[1] RIKEN Systems and Structural Biology Center, Tsurumi, Yokohama 230-0045, Japan
[2] RIKEN Center for Life Science Technologies, Tsurumi, Yokohama 230-0045, Japan
[3] Laboratory for Structural Neuropathology, RIKEN Brain Science Institute, Wako, Saitama 351-0198, Japan
[4] Department of Neuroscience for Neurodegenerative Disorders, Juntendo University Graduate School of Medicine, Tokyo 113-8421, Japan
[5] Laboratory of Structural Neuropathology, Doshisha University Graduate School of Brain Science, 1-3 Tatara Miyakodani, Kyotanabe-shi, Kyoto 610-0394, Japan
[6] Department of Chemistry and Life Science, Kogakuin University, Hachioji, Tokyo 192-0015, Japan
[7] RIKEN Structural Biology Laboratory, Tsurumi, Yokohama 230-0045, Japan

SUPPLEMENTAL FIGURE LEGENDS

SUPLLEMENTAL FIGURE S1. Electron densities (2*F*o−*F*c composite omit map contoured at 1.5 σ) of the N-terminal segments of the mouse and human β4ex structures.

SUPLLEMENTAL FIGURE S2. Full-length blots and gels presented in the main figures.

SUPPLEMENTAL TABLE LEGENDS

SUPPLEMENTAL TABLE S1. Data collection and refinement statistics.

Supplemental Figure S1

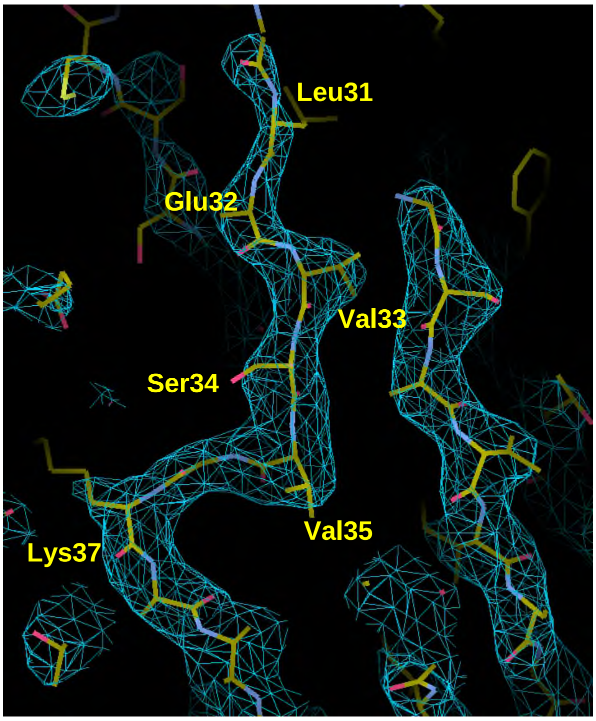

Mouse β4 cubic form

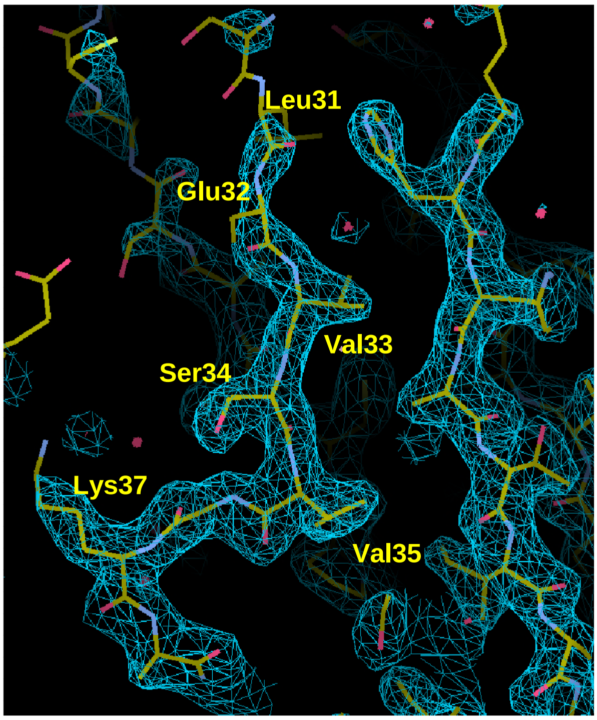

Human β4 hexagonal form

Supplemental Figure S2

Fig. 4

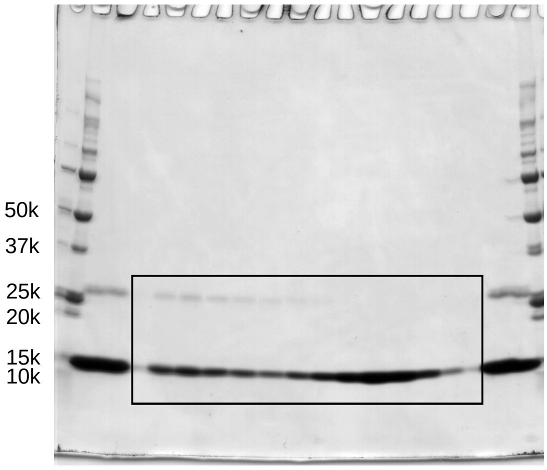

Fig. 5*A*

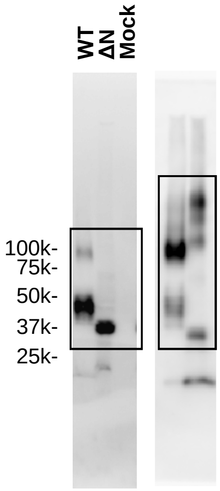

Fig. 5*F*

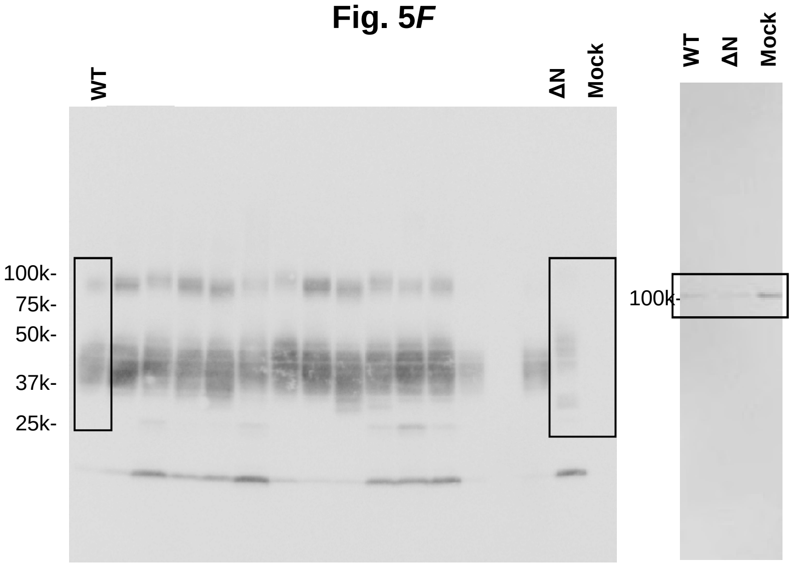

Fig. 6*B*

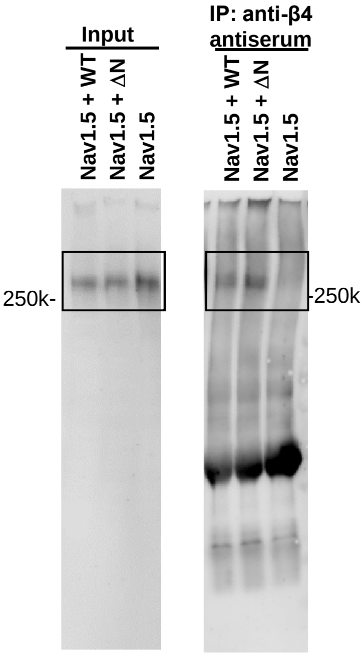

Fig. 6*C*

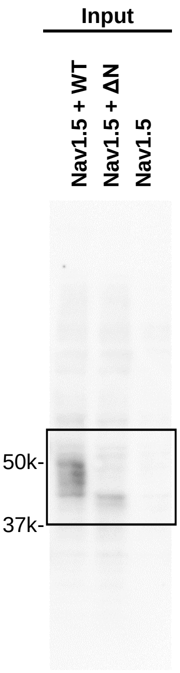

Supplemental Table S1

	Mouse β4 cubic form	Human β4 hexagonal form
**Data collection**		
Space group	*I*23	*P*32
Unit-cell parameters (Å, °)	131.5, 131.5, 131.5, 90, 90, 90	57.1, 57.1, 72.0, 90, 90, 120
Resolution (Å)	100–2.9 (3.00–2.9)	100–2.1 (2.18–2.1)
R_{sym} (%)	8.6 (143.5)	7.3 (47.4)
$I / \sigma (I)$	31.4 (1.8)	32.5 (1.7)
Completeness (%)	99.6 (100)	97.4 (79.4)
Redundancy	7.9 (8.0)	6.4 (3.1)
$CC_{1/2}$	0.999 (0.545)	0.998 (0.846)
**Refinement**		
Resolution (Å)	35.2–2.9 (3.08–2.9)	29.1–2.1 (2.17-2.1)
No. reflections	8526 (1267)	14834 (1045)
R_{work}/ R_{free} (%)	24.6/30.0 (34.6/39.2)	20.4/26.4 (24.5/34.9)
No. atoms		
Protein	1885	1765
Water	0	41
Ligand	6	95
B-factor ($Å^2$)		
Protein	90.4	50.7
Water	-	53.6
Ligand	59.0	53.9
R.m.s. deviations		
Bond lengths (Å)	0.005	0.007
Bond angles (°)	0.978	0.964
Ramachandran plot		
Favored (%)	91.2	97.6
Outliers (%)	0.4	0

Values in parentheses are for the highest-resolution shell.
